# Cystatins 9 and C as a Novel Immunotherapy Treatment That Protects against Multidrug-Resistant New Delhi Metallo-Beta-Lactamase-1-Producing Klebsiella pneumoniae

**DOI:** 10.1128/AAC.01900-17

**Published:** 2018-02-23

**Authors:** Alex J. Holloway, JiehJuen Yu, Bernard P. Arulanandam, Sarah M. Hoskinson, Tonyia Eaves-Pyles

**Affiliations:** a Department of Microbiology and Immunology, University of Texas Medical Branch, Galveston, Texas, USA; b The South Texas Center for Emerging Infectious Diseases, Department of Biology, University of Texas at San Antonio, San Antonio, Texas, USA

**Keywords:** multidrug resistant, New Delhi metallo-beta-lactamase-1-producing K. pneumoniae, antimicrobial, cystatin 9, cystatin C, immunotherapy, immunomodulation, inflammation, pneumonia

## Abstract

Multidrug-resistant (MDR) bacterial pneumonia can induce dysregulated pulmonary and systemic inflammation leading to morbidity and mortality. Antibiotics to treat MDR pathogens do not function to modulate the extent and intensity of inflammation and can have serious side effects. Here we evaluate the efficacy of two human cysteine proteinase inhibitors, cystatin 9 (CST9) and cystatin C (CSTC), as a novel immunotherapeutic treatment to combat MDR New Delhi metallo-beta-lactamase-1 (NDM-1)-producing Klebsiella pneumoniae. Our results showed that mice infected intranasally (i.n.) with a 90% lethal dose (LD_90_) challenge of NDM-1 K. pneumoniae and then treated with the combination of human recombinant CST9 (rCST9) and rCSTC (rCSTs; 50 pg of each i.n. at 1 h postinfection [p.i.] and/or 500 pg of each intraperitoneally [i.p.] at 3 days p.i.) had significantly improved survival compared to that of infected mice alone or infected mice treated with individual rCSTs (*P* < 0.05). Results showed that both of our optimal rCST treatment regimens modulated pulmonary and systemic proinflammatory cytokine secretion in the serum, lungs, liver, and spleen in infected mice (*P* < 0.05). Treatment also significantly decreased the bacterial burden (*P* < 0.05) while preserving lung integrity, with reduced inflammatory cell accumulation compared to that in infected mice. Further, rCST treatment regimens reduced lipid peroxidation and cell apoptosis in the lungs of infected mice. Additionally, *in vitro* studies showed that rCSTs (50 or 500 pg of each) directly decreased the viability of NDM-1 K. pneumoniae. In conclusion, the data showed that rCST9/rCSTC worked synergistically to modulate host inflammation against MDR NDM-1 K. pneumoniae pneumonia, which significantly improved survival. Therefore, rCST9/rCSTC is a promising therapeutic candidate for the treatment of bacterial pneumonia.

## INTRODUCTION

Cystatin 9 (CST9) and cystatin C (CSTC) are small, ∼18-kDa human cysteine proteinase inhibitors and members of the type 2 cystatin superfamily. Members of this cystatin family are found in all body compartments and fluids where matrix metalloproteinases (MMPs) are present (1, 2). MMPs, secreted by immune, epithelial, and endothelial cells, are cysteine proteinases that break down extracellular matrices (ECM) (1, 2). Under normal circumstances, MMPs are controlled by cystatins to regulate the degradation of the ECM, thus maintaining tissue remodeling homeostasis (1–3). Additionally, cystatins differentially regulate various types of lysosomal cathepsins and caspase enzymes to prevent excessive intracellular proteolysis and breakdown of the extracellular matrix (3). Under pathophysiological conditions, such as infection and other disease states, an imbalance between cysteine proteinases and their CST inhibitors can occur. As such, cystatin levels are insufficient to restrain tissue breakdown, which can result in excessive immune cell extravasation to the site of inflammation, leading to direct and secondary organ damage (e.g., systemic inflammatory response syndrome [SIRS] and acute respiratory distress syndrome [ARDS]), and can allow pathogen invasion of healthy tissue (4–7).

When the proteinase-inhibitor balance is restored, CSTs have immunomodulatory effects on the host (e.g., downregulating cysteine proteinases involved in apoptosis and tissue destruction and modulating cytokine secretion as well as immune cell migration). Such functions temper immune responses that would otherwise result in host tissue damage (2, 4, 5). Although the immunomodulatory effects and therapeutic benefits of CSTs remain under investigation, CSTC is the most studied, to date, and has been reported to protect against neurodegeneration (8–10), prevent metastasis of certain cancer cells (11–13), and reduce HIV replication in macrophages (14). Less is known about CST9. We were the first to show the immunomodulatory and antimicrobial effects of recombinant CST9 (rCST9) against pulmonary tularemia (15). Our findings revealed that a single intranasal (i.n.) dose of rCST9 during a pulmonary Francisella tularensis infection significantly improved survival outcomes, increased human macrophage killing of F. tularensis, and diminished F. tularensis viability (15).

Pneumonia and acute lung injury (ALI)/ARDS caused by multidrug-resistant (MDR) pulmonary bacterial pathogens, such as New Delhi metallo-beta-lactamase-1-producing Klebsiella pneumoniae (NDM-1 K. pneumoniae), are a source of high morbidity and mortality in adult and pediatric populations around the world and are a significant contributor to hospital care costs (16–24). The most common organisms that produce NDM-1 are K. pneumoniae and Escherichia coli, but other bacteria can acquire the gene via horizontal gene transfer (25–27). NDM-1 plasmids encode enzymes that render bacteria resistant to a broad spectrum of beta-lactam antibiotics, including the carbapenems (25, 28); as such, these NDM-1^+^ pathogens have been designated “superbugs.” Carbapenems kill bacteria by inhibiting the synthesis of a component of the cell wall membrane. However, bacteria containing the *bla*_NDM-1_ gene produce the NDM-1 enzyme, which hydrolyzes and inactivates carbapenem antibiotics (28, 29). NDM-1 was first identified in a K. pneumoniae isolate from a Swedish patient of Indian origin in 2008 and then detected later in various countries worldwide, including the United States (22–24). The NDM-1-producing bacteria can cause life-threatening lung injury, characterized by dysregulated inflammatory responses leading to (i) excess cytokine secretion; (ii) increased tissue breakdown by MMPs (proteinase-proteinase inhibitor imbalance), with subsequent destruction of the alveolar epithelium and vascular endothelium (30, 31); and (iii) increased permeability of the alveolar wall and accumulation of inflammatory cells (30–32).

Although certain levels of inflammation are required to combat such infections, unrestrained inflammation can worsen an infection and/or disease. Current antimicrobial and/or anti-inflammatory agents typically fail to modulate the extent and intensity of the inflammatory cascade, resulting in an imbalance of immune functions and thereby increasing the risk of secondary infection. We have identified the use of human rCST9 and rCSTC as a novel and effective immunotherapeutic approach to treatment of MDR NDM-1 K. pneumoniae respiratory infection via multifaceted modulation of pathogenic inflammation while promoting beneficial immune responses and directly interfering with pathogen viability. In the present study, we established a murine model of pneumonia caused by NDM-1 K. pneumoniae. Using this model, we determined the optimal efficacy of human rCST9/rCSTC as a countermeasure against pneumonia. The results of these studies establish a novel, potentially therapeutic use for exogenous rCST9/rCSTC in patients suffering from pneumonia and support future studies of these CSTs as a treatment against other pathogenic bacterial lung diseases.

## RESULTS

### Efficacy of rCST9/rCSTC administration post-NDM-1 K. pneumoniae infection.

**FIG 1:**
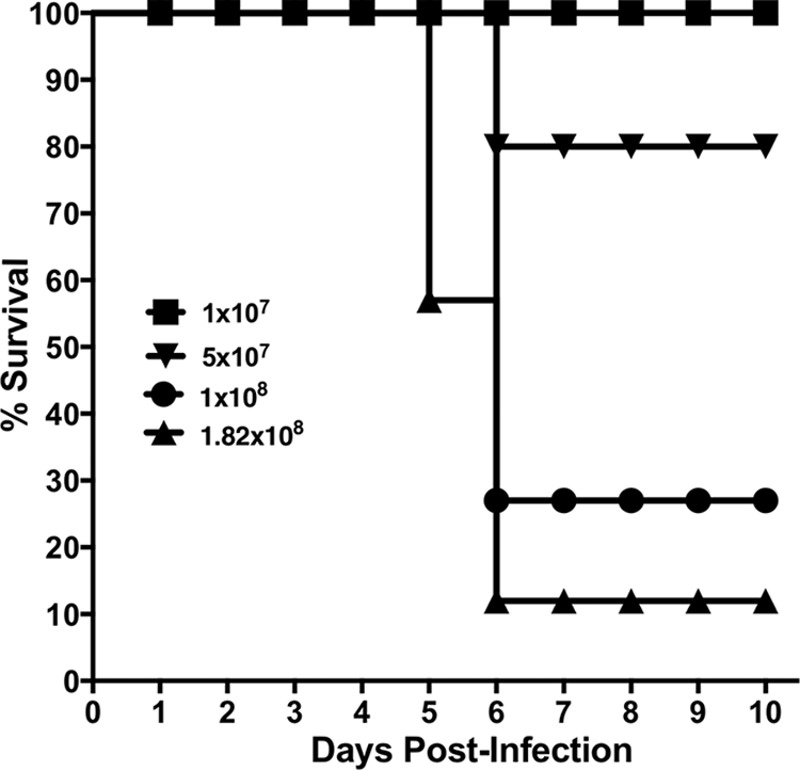
Establishment of LD_90_ for murine model of NDM-1 K. pneumoniae pneumonia. BALB/c mice (*n* = 20 mice/group) were inoculated intranasally (i.n.) with various challenge doses of NDM-1 K. pneumoniae, and their survival was monitored for 10 days. The LD_90_ of K. pneumoniae was 1.82 × 10^8^ CFU per mouse.

We first established the infectious dose of NDM-1 K. pneumoniae that would cause 90% mortality (90% lethal dose [LD_90_]) in BALB/c mice. Mice (*n* = 20 to 30 mice/group) were challenged i.n. with various doses of the clinical isolate NDM-1 K. pneumoniae 2146, and then survival was monitored daily. Using the endpoint estimation method (Reed and Muench), 1.82 × 10^8^ CFU/mouse was calculated as the LD_90_ leading to imminent death by 6 to 7 days postinfection (p.i.) (Fig. 1).

**FIG 2:**
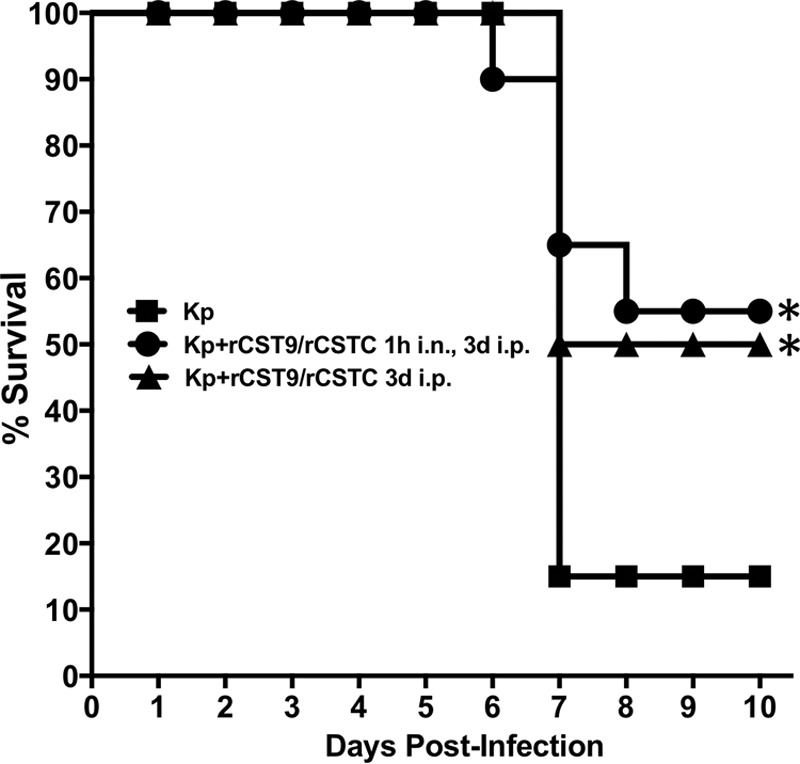
Optimal rCST9/rCSTC treatment regimens afforded protection against NDM-1 K. pneumoniae (Kp) pneumonia. BALB/c mice (*n* = 20 mice/group) were infected i.n. with NDM-1 K. pneumoniae (1.82 × 10^8^ CFU/mouse) and then treated as follows: mice were given an i.n. dose of rCST9/rCSTC (50 pg of each/mouse) at 1 h p.i. followed by 500 pg (each) of rCST9/rCSTC per mouse at 3 days p.i., or mice were administered a single i.p. dose of rCST9/rCSTC (500 pg of each/mouse). Both rCST treatment regimens significantly increased survival compared to that of untreated NDM-1 *K. pneumoniae*-infected mice (*P* < 0.05). Data are presented as means ± SEM, and asterisks signify significant differences (*P* < 0.05).

To establish the optimal CST route, timing, and dosage regimen against MDR NDM-1 K. pneumoniae-induced pneumonia, BALB/c mice (*n* = 10 to 20 mice/group) were infected i.n. at the LD_90_ and treated at various times and doses as depicted in Table 1. Doses included i.n. and/or intraperitoneal (i.p.) treatment with rCST9, rCSTC, or a combination of rCST9 and rCSTC. Mice that were infected with NDM-1 K. pneumoniae but were not treated served as controls. rCST treatments with different routes or doses or as a combined therapy were studied to select the optimal regimen. The results showed that i.n. monotherapy treatment with either rCST9 or rCSTC at 50 or 500 pg delivered at 1 day and/or 3 days p.i. increased survival 5 to 20% compared to the findings with untreated NDM-1 *K. pneumoniae*-infected mice. However, the specific timing and route of the rCST9/rCSTC combination p.i. markedly improved survival of K. pneumoniae-infected mice compared to that of mice given individual treatments (Table 1). Mice treated i.n. with rCST9/rCSTC (50 pg of each) at 1 h p.i. and then given rCST9/rCSTC (500 pg of each) i.p. at 3 days p.i. had significantly improved survival outcomes compared to those for the other groups in the study, except for one additional dosage regimen (Table 1 and Fig. 2) (*P* < 0.05). Interestingly, nearly equivalent survival was observed when a single i.p. dose of rCST9/rCSTC (500 pg/mouse) was given at 3 days p.i. compared to survival rates of untreated NDM-1 *K. pneumoniae*-infected mice (Table 1 and Fig. 2) (*P* < 0.05). These data established the unprecedented synergistic efficacy against NDM-1 *K. pneumoniae*-induced pneumonia of the combined rCSTs given i.n. at 1 h p.i. (50 pg of each CST/mouse) and i.p. at 3 days p.i. or given as a single i.p. dose at 3 days p.i. (500 pg of each CST/mouse). Both of these rCST treatment regimens were used throughout the remainder of the studies. These evaluations led us to select the following abbreviated names for our two optimal rCST treatments: (i) “rCST9/rCSTC 1h i.n., 3d i.p.” represents the dual i.n. and i.p. rCST treatment regimen, and (ii) “rCST9/rCSTC 3d i.p.” signifies the single treatment with rCSTs at 3 days p.i. We used both rCST treatment regimens and these abbreviations throughout the remainder of the studies.

**TABLE 1:** Cystatin treatment regimens to combat NDM-1 K. pneumoniae pneumonia

Treatment	Dose (each) per mouse (pg)	Route	Time(s) of administration		% increased survival of mice infected with K. pneumoniae at the LD_90_
			Preinfection	Postinfection	
rCST9 or rCSTC	50	i.n.	1 h		10 and 20, respectively
	500	i.n.	1 h		10
	50	i.n.		3 days	10
	500	i.n.		1 h and 3 days	10
	50	i.p. then i.p.		1 h and 3 days	5
rCST9 and rCSTC	50, 500	i.n. then i.p.		1 and 3 days	20
	500	i.p. then i.n.		1 and 3 days	10
	500	i.p.		4 days	5
	500	i.p.		5 days	0
	500	i.p.		1 and 3 days	5
	50, 500	i.n. then i.p.		1 and 3 days	25
	50, 500	i.n. then i.p.		1 h and 3 days	38
	500	i.p.		3 days	35

### PEG-rCST9/rCSTC and colistin administration to improve survival outcomes during NDM-1 K. pneumoniae pneumonia.

**FIG 3:**
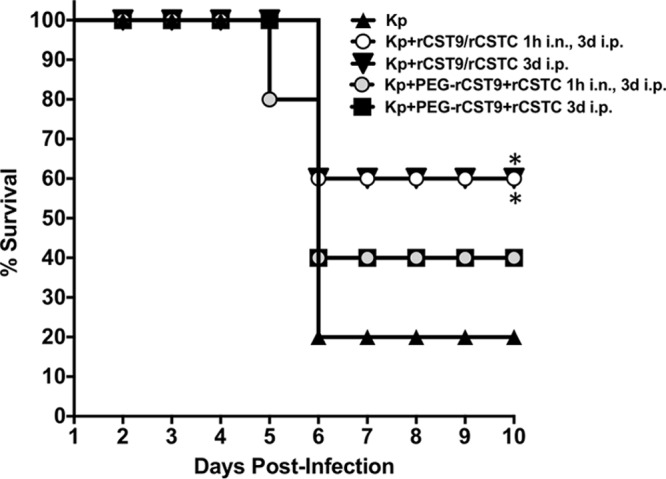
Pegylated rCST9 coadministered with rCSTC did not improve survival outcomes of NDM-1 K. pneumoniae-infected mice. BALB/c mice (*n* = 15 mice/group) were infected i.n. with an LD_90_ challenge with NDM-1 K. pneumoniae and then treated with an i.n. dose of PEG-rCST9/rCSTC (50 pg of each/mouse) at 1 h p.i. followed by 500 pg (each) of PEG-rCST9/rCSTC per mouse at 3 days p.i. or administered a single i.p. dose of PEG-rCST9/rCSTC (500 pg of each/mouse) at 3 days p.i. PEG-rCST9/rCSTC did not improve the survival of infected mice over that seen with rCST9/rCSTC. However, both groups that received the i.p. administration of rCST9 or PEG-rCST9 in combination with rCSTC on day 3 p.i. showed significantly increased survival compared to that of untreated NDM-1 K. pneumoniae-infected mice (*P* < 0.05). Data are presented as means ± SEM, and asterisks signify significant differences (*P* < 0.05).

In an effort to enhance the bioavailability and efficacy of rCSTs in the host, we generated pegylated rCST9 (PEG-rCST9) to evaluate if the combination of PEG-rCST9 and rCSTC would improve survival outcomes against pneumonia. We treated mice challenged with NDM-1 K. pneumoniae at the LD_90_ (*n* = 10 mice/group) with our two optimized rCST dosage regimens (rCST9/rCSTC 1h i.n., 3d i.p. and rCST9/rCSTC 3d i.p.). Parallel groups of infected mice were treated as described above, except that PEG-rCST9 replaced rCST9. Untreated NDM-1 K. pneumoniae-infected mice again served as controls. As shown in Fig. 3, survival outcomes were not improved by pegylation of rCST9. In fact, rCST9/rCSTC-treated mice had a survival rate that was 20% higher with both established treatment regimens than that with PEG-rCST9/rCSTC treatments and significantly higher than that of untreated NDM-1 K. pneumoniae-infected mice (*P* < 0.05).

**FIG 4:**
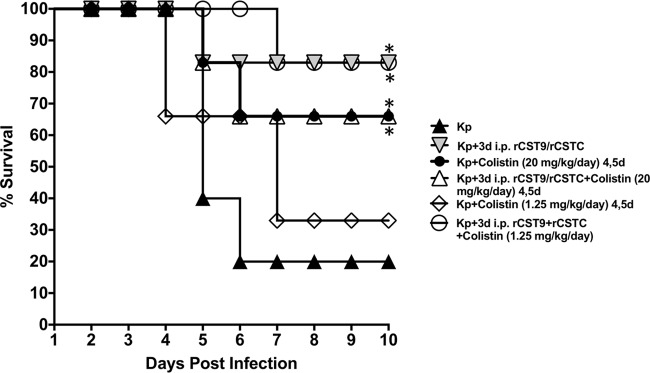
rCST9/rCSTC and colistin polytreatment survival outcomes for mice infected with a lethal pulmonary challenge with NDM-1 K. pneumoniae. BALB/c mice (*n* = 15 mice/group) were infected i.n. with NDM-1 K. pneumoniae (1.82 × 10^8^ CFU/mouse) and then given an i.p. dose of 500 pg (each) of rCST9/rCSTC per mouse at 3 days p.i. The mice were then given colistin (20 mg/kg/mouse or 1.25 mg/kg/day) i.p. 2 times/day (8 h apart) for 2 days, at 4 and 5 days p.i. rCST treatment followed by 20 mg/kg/day of colistin did not improve survival over that with rCST treatment alone. The high colistin dose alone or rCST treatment alone improved survival to a level equal to the survival rate of untreated infected mice (*P* < 0.05). Conversely, the polytreatments of rCSTs and a very low dose of colistin (1.2 mg/kg/day) did improve survival outcomes for infected mice compared to those with colistin treatment alone or those for untreated NDM-1 K. pneumoniae-infected mice (*P* < 0.05). Data are presented as means ± SEM, and asterisks signify significant differences (*P* < 0.05).

Further, we addressed the potential for rCST9/rCSTC treatment to extend the period before successful antibiotic intervention was initiated. For these evaluations, we used suboptimal doses of colistin to determine the extent of protection afforded by rCSTs as well as to minimize side effects/toxicity caused by this antibiotic (*n* = 10 mice/group). The doses, timing, and administration route of colistin were chosen based on efficacy studies of colistin in a mouse model of severe, established MDR pneumonia (33), or a very low dose of colistin was used and then adjusted to suboptimal levels for this study. Mice challenged with NDM-1 K. pneumoniae at the LD_90_ were treated with the rCST9/rCSTC 3d i.p. treatment and then given 2 doses of colistin (20 mg/kg of body weight/day or 1.25 mg/kg/day) on days 4 and 5 p.i. Conversely, rCST treatment administered prior to administration of 1.25 mg/kg/day of colistin at 4 and 5 days p.i. significantly improved survival outcomes for NDM-1 *K. pneumoniae*-infected mice (Fig. 4) (*P* < 0.05). Interestingly, rCST treatment alone confirmed that the rCST treatment led to an unprecedented improvement in survival of NDM-1 *K. pneumoniae*-infected mice compared to that of mice given the low dose of colistin alone and the NDM-1 K. pneumoniae-infected controls (Fig. 4) (*P* < 0.05). These results showed that rCST treatment extended the period before antibiotic intervention was initiated and, importantly, that rCST treatment worked synergistically with a dramatically lower, less toxic dose of colistin to combat pneumonia.

### rCST treatments modulated local and systemic inflammation to protect against NDM-1 K. pneumoniae pneumonia.

**FIG 5:**
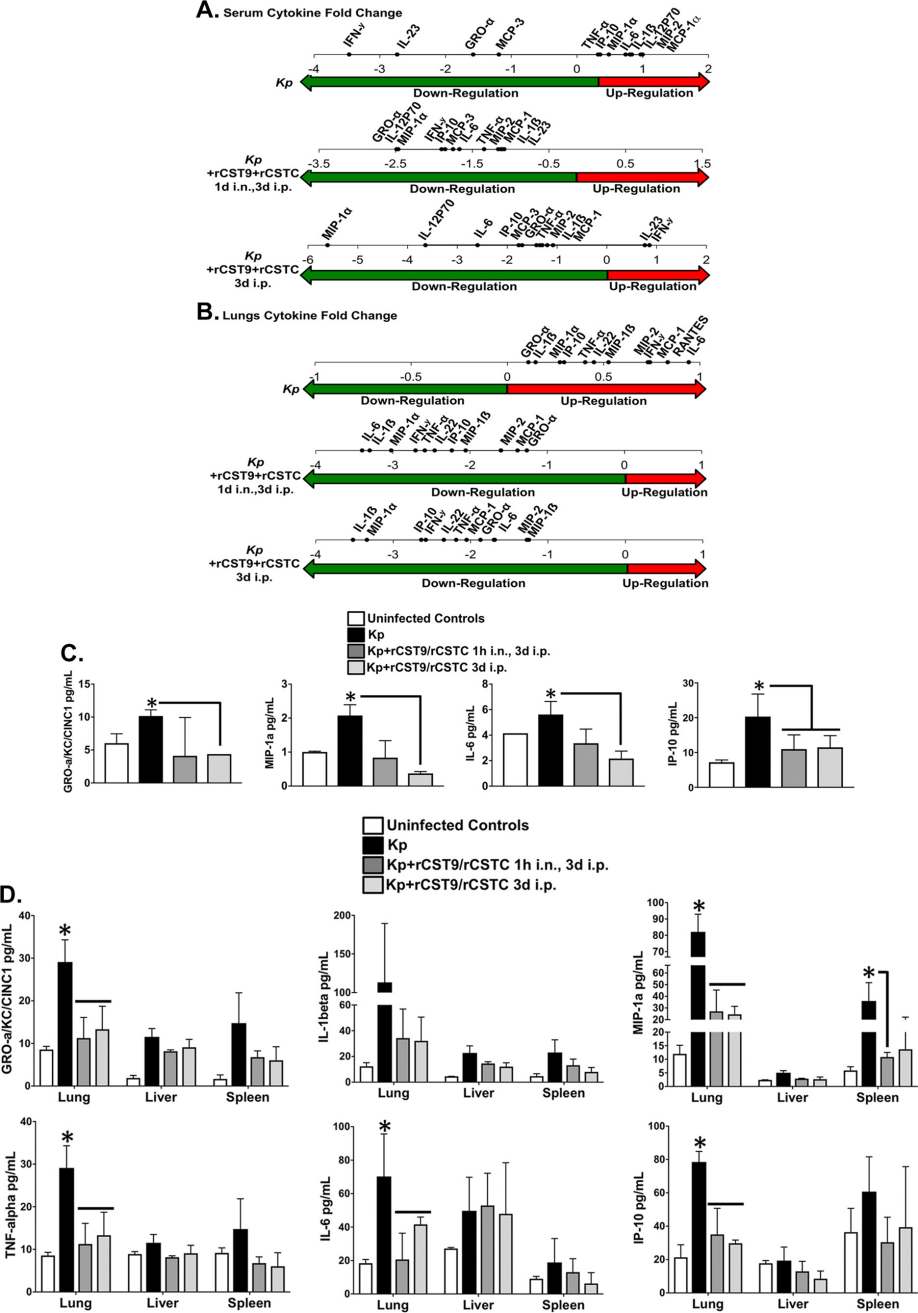

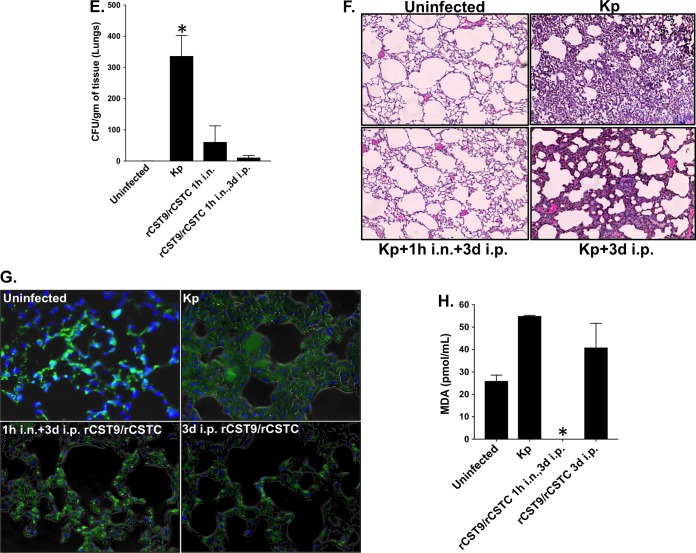
rCST9/rCSTC treatment modulated inflammatory responses and preserved lung integrity in a mouse model of pneumonia. BALB/c mice (*n* = 6 mice/group) were infected i.n. with NDM-1 K. pneumoniae (1.82 × 10^8^ CFU/mouse) and then treated with an i.n. dose of rCST9/rCSTC (50 pg of each/mouse) at 1 h p.i. followed by 500 pg (each) of rCST9/rCSTC per mouse at 3 days p.i. or administered a single i.p. dose of rCST9/rCSTC (500 pg of each/mouse). Sera were collected and lungs, livers, and spleens harvested at 5 days p.i. Fold changes in the overall cytokine levels in the serum (A) and lungs (B) for rCST-treated mice were decreased compared to those for untreated NDM-1 K. pneumoniae-infected mice. Both rCST9/rCSTC treatments modulated cytokine secretion in the serum (C) as well as in all tested organs (D) and significantly reduced the bacterial burden in the lungs (E). (F) Lung histology (H&E staining; magnification, ×40) for the same treated and/or infected mice showed that both rCST treatment regimens minimized lung pathology caused by NDM-1 K. pneumoniae. (G) Further, rCST treatment reduced the number of apoptotic cells compared to that for untreated infected mice. (H) Likewise, MDA detection in the lungs was significantly decreased in rCST-treated infected mice. Graphical data are presented as means and SEM, and asterisks signify significant differences (*P* < 0.05).

We evaluated our two established rCST9/rCSTC treatments for the ability to preserve lung integrity and modulate inflammation in NDM-1 K. pneumoniae-infected mice. Mice (*n* = 6/group) were infected and treated as described above. Uninfected, infected, and rCST monotherapy-treated mice served as comparators. Sera, lungs, livers, and spleens were collected on day 5 p.i. to analyze bacterial load, lung histology, and cytokine profiles. We chose to collect *in vivo* samples on day 5 p.i. because untreated infected mice succumbed to the infection on day 5 to 7 p.i. (Fig. 1). Our previous findings showed that rCST monotherapy treatment did not induce cytokine secretion above that for uninfected controls (data not shown). Both combination rCST treatment regimens effectively downregulated the overall secretion of numerous proinflammatory cytokines in NDM-1 K. pneumoniae-infected mice. Figure 5A and B show fold changes in cytokine levels in the sera and lungs of rCST-treated mice compared to those in untreated NDM-1 K. pneumoniae-infected mice. The sera and lungs of rCST-treated infected mice showed a downregulation, but not elimination, of cytokine secretion compared to that in untreated NDM-1 K. pneumoniae-infected mice (Fig. 5A and B). Interestingly, interleukin-23 (IL-23) and gamma interferon (IFN-γ) were upregulated in the sera of mice treated i.p. with rCST9/rCSTC at 3 days p.i., while these cytokines were downregulated in untreated infected mice (Fig. 5A). IFN-γ and IL-23 are important for controlling K. pneumoniae infections both at the primary site of infection and systemically. Determination of secreted cytokine levels (measured in picograms per milliliter) showed that GRO-α/KC, IL-6, macrophage inflammatory protein 1α (MIP-1α), and IP-10 levels were decreased in the sera of mice in the rCST9/rCSTC 1h i.n., 3d i.p. group. These cytokines were significantly reduced in the rCST9/rCSTC 3d i.p. group compared to those in the untreated infected mice (Fig. 5C) (*P* < 0.05). Likewise, the combination rCST treatments modulated cytokine secretion in the liver, spleen, and, most notably, lungs (GRO-α/KC/CINC1, MIP-1α, tumor necrosis factor alpha [TNF-α], and IL-6) compared to the findings for untreated infected mice (Fig. 5D) (*P* < 0.05). Additionally, both rCST treatment regimens significantly reduced NDM-1 K. pneumoniae burdens in the lungs compared to those for untreated infected mice (Fig. 5E).

Lung histology analysis of the same mice revealed that the rCST9/rCSTC 1h i.n., 3d i.p. group showed a normal, healthy lung architecture equivalent to that in uninfected lungs (Fig. 5F), with no signs of edema or fluid-filled alveoli, consistent with restrained inflammatory cell infiltration. Further, histological examination of lungs from mice treated with a single i.p. dose of rCST9/rCSTC showed a preservation of lung architecture with a moderate accumulation of inflammatory cells and no alveolar wall injury, hemorrhaging, or hyperplasia compared to that in untreated *K. pneumoniae*-infected mice (Fig. 5F). Likewise, rCST treatment markedly reduced cell apoptosis in the lungs of infected mice compared to that in the lungs of untreated infected mice (Fig. 5G). Additionally, lung homogenates were analyzed for cell-damaging lipid peroxidation as determined via malondialdehyde (MDA) quantification. Results showed that the MDA level was decreased when NDM-1 *K. pneumoniae*-infected mice were administered either optimal rCST treatment; however, minimal to no MDA was detected in mice treated with rCST9/rCSTC 1h i.n., 3d i.p. compared to that in all other groups (Fig. 5H). To confirm that the administration of rCST9/rCSTC alone did not induce pulmonary damage, mice were treated i.n. with rCST9/rCSTC (50 pg of each), and then lungs were collected at 30 min, 1 h, and 3 h posttreatment. No measurable levels of MDA above control baselines were detected in the lungs of the rCST9/rCSTC-treated mice at any time point (data not shown).

The culmination of these data showed that rCST9/rCSTC administration to the primary site of infection (i.n.) modulated inflammation, prevented lung damage, and decreased the bacterial burden following NDM-1 K. pneumoniae infection. Moreover, rCST9/rCSTC given i.p. modulated both systemic and lung inflammation.

### rCSTs prevented lung pathology in a murine model of NDM-1 K. pneumoniae pneumonia.

**FIG 6:**
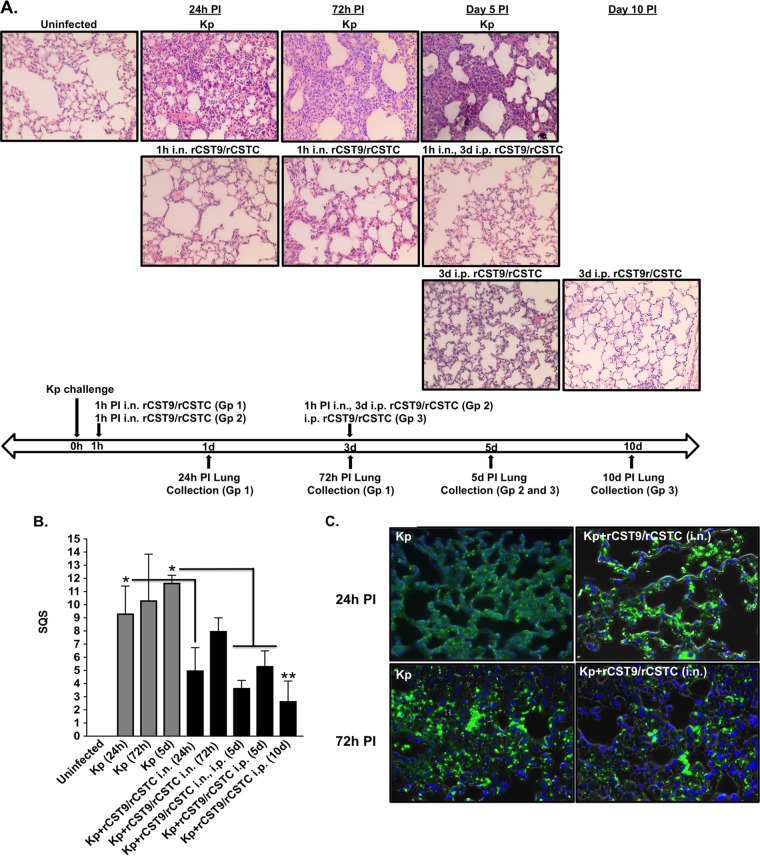
rCST treatments preserved lung integrity and prevented long-term lung damage. BALB/c mice (*n* = 4 mice/group) were infected i.n. with NDM-1 K. pneumoniae (1.82 × 10^8^ CFU/mouse) and then treated with an i.n. dose of rCST9/rCSTC (50 pg of each). The lungs were harvested at 24 h and 72 h p.i. A parallel group of mice were infected and treated i.n./i.p. or i.p. with rCST9/rCSTC as described herein, and then lungs were harvested at 5 and 10 days p.i. Serial sections of the lung were analyzed for histology (magnification, ×40) and apoptosis by using the TUNEL assay with DAPI to stain cell nuclei. (A) The i.n. administration of rCST9/rCSTC to infected mice markedly diminished immune cell infiltration into the lungs and edema at 24 h and 72 h p.i. compared to the high cellularity and signs of hemorrhaging and edema in the lungs of untreated infected mice. Further, lungs from mice treated with our two optimal rCST9/rCSTC treatments, obtained at 5 and 10 days p.i., showed prevention of long-term lung damage and resolution of inflammation. (B) Histopathological scoring of the lungs (0, no significant changes; 1, slight damage; 2, mild to moderate damage; 3, moderate to severe damage; and 4, severe damage) for three categories. The results showed that cystatin treatments significantly decreased lung damage compared to that at corresponding time points for untreated infected mice. Lungs from mice receiving rCSTs at 3 days p.i. and lungs collected from survivors at 5 and 10 days p.i. had mild to no damage, in contrast to the lungs of untreated infected mice (*, *P* < 0.05; **, *P* < 0.01). The scoring results are expressed as SQS (means and SEM). (C) Likewise, lungs from the same rCST-treated and infected groups showed markedly fewer apoptotic cells at 24 and 72 h p.i. All images are representative of the analysis of 4 to 6 sections per mouse.

To determine the early and late effects of rCST9/rCSTC in the lungs of mice infected with NDM-1 K. pneumoniae at the LD_90_ (*n* = 4/group), animals were treated i.n. with rCST9/rCSTC (50 pg of each) at 1 h p.i. Lung tissues were collected at 24 h and 72 h. Parallel groups of mice received the rCST9/rCSTC 1h i.n., 3d i.p. regimen, followed by lung tissue collection at 5 or 10 days p.i. (Fig. 6A). Blind observers evaluated lung histopathology by using a semiquantitative score (SQS) that represents changes in hematoxylin-eosin (H&E)-stained lung tissue (Fig. 6B). A terminal deoxynucleotidyltransferase-mediated dUTP-biotin nick end labeling (TUNEL) assay was also performed on serial sections of lung tissue to detect apoptotic cells. Results shown are representative of the culmination for each individual group. Histological (Fig. 6A and B) and TUNEL (Fig. 6C) analyses of untreated NDM-1 *K. pneumoniae*-infected mice revealed that lung pathology deteriorated significantly as the disease progressed over time, as shown by the lung histopathological examination results (Fig. 6A) and SQS (Fig. 6B) (*P* < 0.05). At 24 h p.i., the lungs exhibited high cellularity caused by immune cell infiltrates and alveolar hemorrhage, as evidenced by the presence of red blood cells (RBCs) (Fig. 6A). By 72 h and 5 days p.i., lungs showed excessive immune cell accumulation, signs of hemorrhaging, and edema as well as destruction of the alveolar architecture (Fig. 6A), which earned these lungs significantly higher SQS than those for lungs of rCST-treated infected mice (Fig. 6B) (*P* < 0.05). Likewise, in serial sections, apoptotic cells were prominent at 24 h p.i. and increased over time (72 h and 5 days p.i.) in the lungs of untreated infected mice (Fig. 6A to C). In contrast, minimal to no pathological changes were observed in lungs collected at 24 and 72 h p.i. from mice receiving i.n. administration of 50 pg (each) rCST9/rCSTC at 1 h p.i., with modest immune cell accumulation at 72 h p.i. (Fig. 6A), resulting in significantly lower SQS for the 24-h rCST-treated infected lungs than those for lungs of untreated infected mice (Fig. 6B) (*P* < 0.05). Lung sections from the same mice showed markedly fewer apoptotic cells at 24 h and 72 h p.i. (Fig. 6C). By 5 days p.i., animals receiving the rCST9/rCSTC 1h i.n., 3d i.p. or rCST9/rCSTC 3d p.i. regimen showed minimal immune cell filtrates/congestion with normal lung architecture and no evidence of long-term damage (Fig. 6A). As such, the 5 day p.i. rCST-treated mouse lungs had significantly lower SQS than those for untreated infected lungs (Fig. 6B) (*P* < 0.05). There were no survivors in the NDM-1 K. pneumoniae-infected group by 8 days p.i. At 10 days p.i., lungs from mice that received i.n. and/or i.p. rCST treatment showed a complete resolution of inflammation, with no residual signs of damage (Fig. 6A) and with significantly lower SQS than those for untreated infected mice (Fig. 6B) (*P* < 0.01). These results demonstrated that rCST treatment modulated a pulmonary inflammation that promoted manageable, transient inflammatory responses to induce pathogen clearance without affecting lung morphology or function.

### Antimicrobial activity of rCST9/rCSTC against NDM-1 K. pneumoniae.

**FIG 7:**
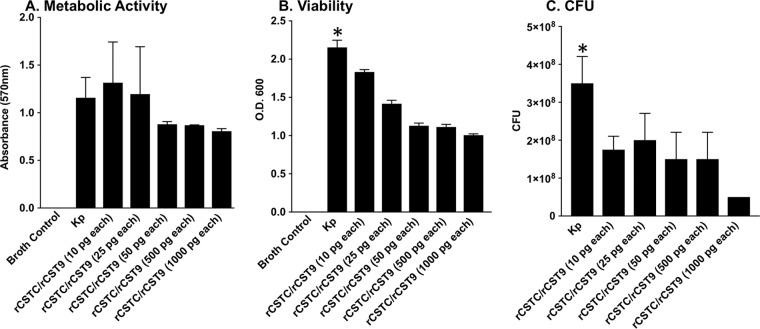
Antimicrobial properties of rCST9/rCSTC against NDM-1 K. pneumoniae. rCST9/rCSTC inhibited the metabolic activity and growth of NDM-1 K. pneumoniae. The 50-, 500-, and 1,000-pg doses of rCST9/rCSTC decreased metabolic activity (A), bacterial replication (B), and growth (C) of NDM-1 K. pneumoniae (1 × 10^6^ CFU/ml) following 6 h of incubation. Data are presented as means and SEM, and asterisks signify significant differences (*P* < 0.05 compared to all other groups).

Past studies also revealed a direct antimicrobial activity of rCSTs. To address this directly, PrestoBlue was used to determine the viability of rCST9/rCSTC-treated NDM-1 K. pneumoniae based on the reagent's rapid reduction by metabolically active bacteria. Our findings revealed that 50, 500, or 1,000 pg (each) of rCST9/rCSTC decreased the viability/metabolic activity of 1 × 10^6^ CFU/ml of NDM-1 K. pneumoniae during 6 h of incubation compared to that of untreated NDM-1 K. pneumoniae and NDM-1 K. pneumoniae incubated with 10 or 25 pg (each) of the rCSTs (Fig. 7A) (*P* < 0.05). Further, at 6 h postincubation, optical density (OD) readings and CFU determinations showed bacterial growth inhibition of NDM-1 K. pneumoniae incubated with all tested doses of rCSTs compared to the growth of untreated NDM-1 K. pneumoniae (Fig. 7B and C) (*P* < 0.05). The most substantial decreases in bacterial growth occurred with 50, 500, and 1,000 pg (each) of rCSTs compared to those with the lower doses of 10 and 25 pg (each) of rCSTs (Fig. 7A to C). These results showed that rCST9/rCSTC directly decreased the viability and growth of NDM-1 K. pneumoniae.

## DISCUSSION

The ability of bacterial organisms to acquire MDR genes is on the rise, making infections caused by MDR pathogens difficult to treat with traditional antibiotics. Therefore, the development of novel, effective therapeutic inventions for these deadly infections is imperative. Here we identified two human cysteine proteinase inhibitors, known as cystatin 9 and cystatin C, as immunomodulators of inflammation caused by deadly pathogens. The present study showed that rCST9 and rCSTC worked synergistically, and in a multifaceted manner, to modulate excessive, damaging inflammatory responses in pneumonia caused by MDR NDM-1 K. pneumoniae. We established the therapeutic efficacy by using two different rCST9/rCSTC treatment regimens in mice challenged with MDR NDM-1 K. pneumoniae at the LD_90_. Postinfection rCST treatment at the primary site of infection (lungs) as well as a single systemic treatment (i.p.) enhanced bacterial clearance and significantly improved survival. Further, we found that our established rCST9/rCSTC treatment regimens were not toxic or harmful to the host.

CSTs were initially identified as cysteine proteinase inhibitors that restrain lysosomal protein degradation and tissue remodeling (1–3). During infections and/or disease states, a proteinase-inhibitor imbalance can develop, leading to dysregulated inflammation and tissue damage (1–5). Recent findings showed that exogenous administration of certain CSTs prevented metastasis of certain cancer cells (6, 11–13), enhanced clearance of HIV by macrophages (14), and prevented degeneration of brain neurons (8–10). However, little has been reported on the role of CSTs during bacterial infection. We were the first investigative team to report that a single i.n. dose of rCST9 (50 pg) attenuated the pathophysiological effects of the deadly human pathogen F. tularensis, resulting in clearance of the pulmonary infection and leading to remarkable improvements in survival rates (15). The success of these studies led us to examine the protective effects of rCST9 in an experimental mouse model of pneumonia.

Initially, we began these experiments by using rCSTC as a comparative control for rCST9 and then unexpectedly discovered that treatment with the combination of rCST9 and rCSTC worked synergistically to combat deadly MDR K. pneumoniae pneumonia. Our efficacy studies determined that low doses and as few as one treatment of rCST9/rCSTC significantly modulated otherwise excessive cytokine secretion to a beneficial level and enhanced bacterial clearance, allowing the host to successfully fight and ultimately resolve the infection, resulting in an unprecedented improvement in survival. Surprisingly, systemic administration of a single i.p. dose of rCST9/rCSTC (500 pg [each]) after the establishment and systemic dissemination (3 days p.i.) of the infection remarkably improved survival outcomes, to levels that were equivalent to those with the dual i.n./i.p. treatment regimen. Moreover, the single rCST9/rCSTC dose tempered damaging pulmonary and systemic cytokine secretion, which decreased lung pathology (e.g., MDA and apoptosis). In fact, in rCST-treated mice, the lung histology was normal, with no signs of long-term damage, by 10 days p.i.

To capitalize on the protective effects of the small, optimal doses of rCSTs, we pegylated rCST9 in an effort to potentially improve its bioavailability in NDM-1 *K. pneumoniae*-infected mice. Pegylation is a method of covalently attaching polyethylene glycol to a target small molecule to improve therapeutic efficacy and pharmacokinetics by enhancing the bioavailability, stability, and half-life of the small-molecule drug *in vivo* (34, 35). Despite successful pegylation, coadministered PEG-rCST9 and rCSTC actually gave decreased survival outcomes. These results are consistent with our testing of higher or more frequent rCST9/rCSTC doses, which did not improve survival against NDM-1 K. pneumoniae pneumonia (data not shown). Additional studies of the PEG-rCST9/rCSTC regimen at lower doses or different combinations of doses will be required to fully appreciate the impact of pegylation. The current findings indicate that the low-dose, two-route delivery course is optimal and provide a solid foundation for subsequent pharmacokinetic and pharmacodynamic studies to advance the development of these CSTs as immunotherapeutic interventions against pneumonia.

Treatment of pneumonia attributed to NDM-1 K. pneumoniae remains challenging because of the acquired resistance to most commercially available antibiotics as well as the pathogen's ability to survive for extended periods on environmental surfaces, both of which are factors that enhance person-to-person transmission (16, 17, 20, 21, 24). Combination antibiotic therapy is routinely implemented to treat NDM-1 K. pneumoniae pneumonia, but antibiotic polytreatments are typically accompanied by serious side effects due to the high doses necessary to fight the infection. The NDM-1 K. pneumoniae strain used in our studies is sensitive to colistin, which is a mixture of polymyxins, specifically polymyxin E, that is considered a last-resort treatment for infections with MDR Gram-negative pathogens because of kidney toxicity (36). Therefore, we used our optimal rCST treatments accompanied by 20 mg/kg/day or 1.25 mg/kg/day of colistin to determine the extent of protection afforded by rCSTs before antibiotic intervention. Administration of 20 mg/kg/day of colistin 2 times/day for 2 days following optimal rCST treatments did not increase survival outcomes over those with the single low dose of rCST alone. However, rCST treatment given prior to the administration of a low, suboptimal dose of 1.25 mg/kg/day of colistin on days 4 and 5 p.i. significantly improved survival outcomes compared to those with colistin alone. Our results showed that the administration of suboptimal antibiotic therapy allowed enhanced efficacy in the presence of rCSTs that will likely minimize side effects/toxicity caused by the antibiotic; however, additional studies will be required to address this question. It is important that neither of the antibiotic doses gave protection surpassing that afforded by rCST monotherapy.

Finally, our findings revealed that 50, 500, and 1,000 pg (each) of rCST9/rCSTC directly inhibited NDM-1 K. pneumoniae metabolic activity and growth. Similarly, we previously reported that rCST9 directly decreased the viability and virulence of the deadly human pathogen F. tularensis Schu 4. Proteomic analysis of F. tularensis exposed to rCST9 showed that the cysteine proteinase inhibitor markedly diminished or eliminated key metabolic proteins in the pathogen, thereby preventing nutritional processing and energy production (15). rCST9 also interfered with the cell wall synthesis of F. tularensis (15). Therefore, rCST9 weakened the pathogen and rendered it more susceptible to killing by the host. Although we have not performed proteomic analyses of rCST9/rCSTC-treated NDM-1 K. pneumoniae, it is reasonable to hypothesize that the combination of rCST9 and rCSTC affected this pathogen in a similar fashion. Although 1,000 pg (each) of the rCSTs inhibited NDM-1 K. pneumoniae metabolic activity and growth nearly equivalently to the results with our optimal rCST *in vivo* doses of 50 and/or 500 pg, the 1,000-pg rCST dose did not provide significant protection in our murine model of pneumonia (data not shown). However, in correlation with our current findings, it was reported that a small peptide derivative of CSTC blocked the growth of group A streptococci both *in vitro* and *in vivo* (37). It has also been shown that CSTC decreases the intracellular replication of various viruses, such as poliovirus (38), herpes simplex virus (HSV) (39), and HIV (14). Likewise, CSTC in combination with IFN-γ led to reduced numbers of Leishmania parasites, enhanced nitric oxide generation, and the conversion of Th_2_ to Th_1_ responses, resulting in the elimination of the parasite (40). To our knowledge, this report is the first to describe the effects of rCST9 (15) and/or rCSTC on deadly MDR bacterial pathogens both *in vitro* and *in vivo*.

Due to the numerous strains of rapidly evolving MDR pathogens, there is an urgent need to develop alternative therapeutic agents to treat infections with these deadly human pathogens. Our findings reveal the multifaceted, synergistic immunoregulatory functions of rCST9/rCSTC. Here we showed that exogenous coadministration of human rCST9/rCSTC preserved lung integrity, modulated local and systemic cytokine secretion, and enhanced antibacterial immune responses and bacterial clearance of MDR NDM-1 K. pneumoniae pneumonia. While we take caution against overinterpreting the results of rCST9/rCSTC treatment as a countermeasure for pneumonia, our findings showed that a single, low-dose administration of rCST9/rCSTC afforded unprecedented protection without toxic side effects. We are currently performing pharmacokinetic and pharmacodynamic analyses of rCST9/rCSTC *in vivo* as well as toxicity evaluation by using a high-throughput drug screening cell culture system. Our findings substantiate that rCST9/rCSTC provides broad-spectrum protection against pneumonia caused by MDR NDM-1 K. pneumoniae and modulates inflammation in multifaceted and unpredicted ways.

## MATERIALS AND METHODS

### Human recombinant CSTC and CST9.

Recombinant human cystatin C was purchased from R&D Systems (Minneapolis, MN), and rCST9 was purchased from American Research Products, Inc. (Waltham, MA).

### Bacterium preparation.

The MDR NDM-1-producing Klebsiella pneumoniae strain BAA-2146 was purchased from ATCC and expanded for 18 h in 10 ml of brain heart infusion (BHI) broth with shaking at 37°C. The overnight culture was pelleted by centrifugation and then suspended in phosphate-buffered saline (PBS). Serial dilutions were performed to obtain the desired concentration. Tenfold dilutions were plated on BHI agar to confirm the experimental dosage.

### Pegylation of rCST9.

In order to generate an N-terminally monopegylated (PEG) human rCST9 protein, purified rCST9 was incubated with 20-kDa methoxy-PEG-propionaldehyde (M-ALD-20K; Jenkem Technology, Beijing, China) and sodium cyanoborohydride (Sigma-Aldrich, St. Louis, MO) at a molar ratio of 1:8:80 in 50 mM sodium acetate buffer (pH 4.5). After 46 h of incubation at room temperature, the mixture was loaded onto a Superdex 75 column (1.6 cm × 60 cm; GE Healthcare, USA) equilibrated with Hanks balanced salt solution (HBSS). Proteins were subsequently eluted and fractionated by using HBSS at a flow rate of 2 ml/min and detected by measuring the absorbance at 280 nm. Fractions containing pegylated rCST9 were further identified, and protein content was profiled by SDS-PAGE analysis.

### Mouse model of pneumonia and CST treatments.

Eight-week-old female BALB/c mice weighing between 21 and 24 g (Jackson Laboratories) were housed in an Association for Assessment and Accreditation of Laboratory Animal Care (AAALAC)-approved housing facility and permitted to adjust to their environment for 7 days prior to procedures, receiving free access to food and water throughout the study. All procedures were approved by the University of Texas Medical Branch IACUC and performed humanely, with minimal suffering. We established an LD_90_ model of pneumonia by anesthetizing mice (*n* = 15 to 20 mice/group) with sodium pentobarbital and then challenging them with an i.n. dose of 1.82 × 10^8^ CFU/mouse of NDM-1 K. pneumoniae as previously described (15, 41). At 1 h p.i., mice were administered an i.n. dose of rCST9/rCSTC (50 pg of each), and then this group of mice was given an i.p. injection of rCST9/rCSTC (500 pg of each) at 3 days p.i. A parallel group of infected mice received only a single i.p. dose of rCST9/rCSTC, PEG-rCST9/rCSTC (500 pg of each), or PEG-rCST9 (500 pg) alone at 3 days p.i. NDM-1 K. pneumoniae-infected mice alone or uninfected mice served as controls. Survival was monitored for up to 15 days posttreatment. Additional groups (*n* = 4 mice/group) of infected and CST-treated mice were euthanized at selected time points (24 h, 72 h, 5 days, and/or 10 days p.i.) to harvest lungs. Additional groups were treated i.n. with rCSTs (50 pg of each) as described above, but they were euthanized at 30 min, 1 h, and 3 h to collect lungs for the quantification of the lipid peroxidase by-product malondialdehyde (MDA) as described below.

Additional groups of mice (*n* = 15 mice/group) were treated with the optimal CST treatment regimens of an i.n. dose of rCST9/rCSTC (50 pg of each) followed by an i.p. injection of rCST9/rCSTC (500 pg of each) at 3 days p.i. or a single i.p. dose of rCST9/rCSTC (500 pg of each). At 4 days p.i., mice were given 2 separate i.p. injections of colistin (colistimethate sodium; JHP Pharmaceuticals, LLC) (20 mg/kg/mouse or 1.25 mg/kg/day) 8 h apart for 2 days. Survival was monitored for 15 to 20 days.

### Lung histology, apoptosis, lipid peroxidation, and bacterial burden.

Following collection, organs were weighed, and then a small portion of the lungs was fixed and processed for H&E staining. A semiquantitative scoring system was employed for scoring of the lung sections collected at 24 h, 72 h, 5 days, and 10 days p.i. (Fig. 6A and B). The entire lung section for each condition was analyzed according to the following categories: structural abnormalities/congestion, hemorrhaging, and cellularity. Lung sections for each condition were analyzed in triplicate for three individual subjects. Each lung section was given a score ranging from 0 to 4 for each of the three categories, as follows: 0, no significant changes; 1, slight damage; 2, mild to moderate damage; 3, moderate to severe damage; and 4, severe damage. The semiquantitative score (SQS) is expressed as the sum of the scores for all three categories. The scoring results are expressed as SQS (mean ± standard error of the mean [SEM]).

Apoptotic cells in parallel lung sections were identified by a TUNEL assay using an *in situ* cell death detection kit (Trevigen) per the manufacturer's instructions. Nuclei were stained with SlowFade Diamond AntiFade mounting reagent with DAPI (4′,6-diamidino-2-phenylindole) (Invitrogen). Remaining lung materials were homogenized in 1 ml of PBS. Aliquots of lung tissue homogenates were analyzed via an MDA assay kit (Cell Biolabs Inc.), used according to the manufacturer's instructions, to detect tissue damage induced by oxidative stress. For each lung, 10% (100 μl) of the gravity-clarified homogenate was plated on BHI agar to determine the bacterial burden. Bacterial counts were calculated and expressed as numbers of CFU per gram of tissue.

### Cytokine profile analysis and enzyme-linked immunosorbent assay (ELISA) kits.

Homogenized tissue supernatants and serum (50-μl samples) were analyzed by use of a ProcartaPlex mouse cytokine/chemokine kit (Affymetrix) to quantify cytokine production. Samples were processed per the manufacturer's instructions on a Bio-Plex200 instrument (Bio-Rad).

### *In vitro* bacterial viability and growth assay.

As a measure of NDM-1 K. pneumoniae viability, aliquots were treated with PrestoBlue cell viability reagent (Invivogen) following exposure to CSTs. Briefly, 1 × 10^6^ CFU/ml of NDM-1 K. pneumoniae was incubated with 10, 25, 50, 500, or 1,000 pg (each) of rCST9/rCSTC at 37°C for 6 h. Following incubation, 10 μl of PrestoBlue reagent was added to each sample and incubated for 1 h before quantification of cell viability via measurement of the absorbance at 570 to 600 nm (Epoch instrument; Bio-Tek). Parallel aliquots of rCST-treated cultures or untreated cultures were used to determine the optical density at 600 nm (OD_600_) of the resulting bacterial cultures via spectrophotometry. Additionally, 100-μl aliquots of cultures were plated on BHI plates to quantify CFU following an overnight incubation at 37°C. These studies were performed per a study by Coban (42) and according to CLSI recommendations.

### Statistical analysis.

Where appropriate, results are reported as means ± SEM for two or three independent experiments. Analysis of numerical data was performed by one-way analysis of variance (ANOVA) and Student's *t* test, using Prism v7.0c software (GraphPad, San Diego, CA). Survival data were analyzed by log rank analyses with Welch's corrections, using Prism software (GraphPad). Differences were considered statistically significant if the *P* value was <0.05.
